# Spontaneous and Acetylcholine Evoked Calcium Transients in the Developing Mouse Utricle

**DOI:** 10.3389/fncel.2019.00186

**Published:** 2019-05-07

**Authors:** Holly A. Holman, Lauren A. Poppi, Micah Frerck, Richard D. Rabbitt

**Affiliations:** ^1^Department of Biomedical Engineering, University of Utah, Salt Lake City, UT, United States; ^2^School of Biomedical Science and Pharmacy, Hunter Medical Research Institute, University of Newcastle, Newcastle, NSW, Australia; ^3^Neuroscience Program, University of Utah, Salt Lake City, UT, United States; ^4^Otolaryngology-Head and Neck Surgery, University of Utah, Salt Lake City, UT, United States

**Keywords:** utricle, calcium, hair cell, supporting cell, neuron, GCaMP5G

## Abstract

Spontaneous calcium transients are present during early postnatal development in the mouse retina and cochlea, and play an important role in maturation of the sensory organs and neural circuits in the central nervous system (CNS). It is not known whether similar calcium transients occur during postnatal development in the vestibular sensory organs. Here we demonstrate spontaneous intracellular calcium transients in sensory hair cells (HCs) and supporting cells (SCs) in the murine utricular macula during the first two postnatal weeks. Calcium transients were monitored using a genetically encoded calcium indicator, GCaMP5G (G5), at 100 ms-frame^−1^ in excised utricle sensory epithelia, including HCs, SCs, and neurons. The reporter line expressed G5 and tdTomato (tdT) in a Gad2-Cre dependent manner within a subset of utricular HCs, SCs and neurons. Kinetics of the G5 reporter limited temporal resolution to calcium events lasting longer than 200 ms. Spontaneous calcium transients lasting 1-2 s were observed in the expressing population of HCs at birth and slower spontaneous transients lasting 10-30 s appeared in SCs by P3. Beginning at P5, calcium transients could be modulated by application of the efferent neurotransmitter acetylcholine (ACh). In mature mice, calcium transients in the utricular macula occurred spontaneously, had a duration 1-2 s, and could be modulated by the exogenous application of acetylcholine (ACh) or muscarine. Long-lasting calcium transients evoked by ACh in mature mice were blocked by atropine, consistent with previous reports describing the role of muscarinic receptors expressed in calyx bearing afferents in efferent control of vestibular sensation. Large spontaneous and ACh evoked transients were reversibly blocked by the inositol trisphosphate receptor (IP_3_R) antagonist aminoethoxydiphenyl borate (2-APB). Results demonstrate long-lasting calcium transients are present in the utricular macula during the first postnatal week, and that responses to ACh mature over this same time period.

## Significance Statement

*Here, we demonstrate the presence of spontaneous calcium transients in sensory hair cells and supporting cells of the developing mouse utricle, prior to the onset of sensing linear gravito-inertial acceleration. Based on these observations, spontaneous calcium activity in the utricle during development is hypothesized to play a role in maturation of vestibular circuits, similar to the role of spontaneous calcium activity in other sensory organs including the retina and cochlea. We further demonstrate that calcium transients can be modulated by the neurotransmitter acetylcholine by postnatal day 5, thus demonstrating a mechanism hypothesized to close the loop between the sensory organ and the central nervous system during maturation of vestibular pathways*.

## Introduction

During development, immature vestibular sensory epithelia include hair cells (HCs), supporting cells (SCs), and undifferentiated precursor cells. In mice, this active developmental period continues during the first two postnatal weeks (Dechesne, 1986; Desmadryl et al., 1986; Burns et al., 2012; Gao et al., 2016). Unique afferent synaptic contacts distinguishing type I vs. type II hair cells (Lysakowski et al., 2011; Warchol et al., 2019) are established during this period. Immature afferent nerve endings either extend to form calyx nerve terminals enveloping the basolateral surfaces of flask-shaped type I hair cells or extend to form bouton terminals on cylindrically-shaped type II hair cells. Evidence suggests that postnatal differentiation of supporting cells adds significant numbers of hair cells to the utricular macula during this period, primarily type II (Warchol, 2007; Kim et al., 2011; Burns and Stone, 2017; Warchol et al., 2019). Gross morphological development of synapses and hair cells precedes mature physiological function as demonstrated by the appearance of hair bundles before mechanoelectrical transduction (MET), and the appearance of afferent calyces surrounding type I hair cells before mature ionic currents (Rüsch et al., 1998; Géléoc and Holt, 2003). The maturation of action potential discharge rate, regularity of discharge inter-spike intervals, and sensitivity to physiological stimulation follows a similar time course as hair cells and synapses reaching mature properties near postnatal day 10 (Curthoys, 1983; Desmadryl and Sans, 1990). During this period, spontaneous bursts of action potentials occur in vestibular ganglion neurons in the absence of gravito-inertial stimulation, thereby providing inputs to vestibular pathways in the central nervous system (CNS). At the same time, efferent synaptic contacts on HCs and afferent nerves are maturing in the vestibular sensory epithelium providing a route for the CNS to modulate excitability and action potentials (Favre and Sans, 1977, 1978; Desmadryl and Sans, 1990; Sans and Scarfone, 1996; Lysakowski, 1999; Kharkovets et al., 2000; Demêmes et al., 2001; Hurley et al., 2006; Sienknecht et al., 2014). How spontaneous vestibular afferent nerve activity is generated, if it is modulated during postnatal development by efferent action, and what role it might play in establishing functional vestibular physiology are as yet unknown.

Like vestibular ganglion neurons, cochlear spiral ganglion neurons in mice exhibit spontaneous action potentials at birth. Spiral ganglion activity occurs before the onset of hearing, and is important to establish tonotopy in the CNS and functional hearing (Lippe, 1994; Kotak and Sanes, 1995; Kros et al., 1998; Glowatzki and Fuchs, 2000; Beutner and Moser, 2001; Tritsch et al., 2007; Sendin et al., 2014). In early postnatal mouse development, spontaneous firing in spiral ganglion neurons is driven by calcium action potentials in auditory inner hair cells (IHCs) (Lippe, 1994; Beutner and Moser, 2001; Glowatzki and Fuchs, 2002; Marcotti et al., 2003; Tritsch et al., 2010; Johnson et al., 2011; Jones and Jones, 2011). Hair cell activity is spontaneous, but modulated by purinergic signaling from supporting cells in Kölliker's organ and by cholinergic signaling from olivocochlear efferent neurons (Zhao et al., 2005; Roux et al., 2011; Zhu and Zhao, 2012; Johnson et al., 2013). Blocking this activity disrupts the fine synaptic organization at all levels in the auditory pathway (Hashisaki and Rubel, 1989; Sanes and Siverls, 1991; Kotak and Sanes, 1995; Rubel and Fritzsch, 2002; Cao et al., 2008; Seal et al., 2008). In the auditory system, calcium currents and intracellular signaling established developmentally are essential substrates of this crucial process (Chabbert, 1997; Kros et al., 1998; Glowatzki and Fuchs, 2000; Marcotti et al., 2003; Katz et al., 2004). Like cochlear IHCs, cells in immature vestibular epithelia express the machinery required to generate spontaneous calcium-dependent activity (Marcotti et al., 2003; Levic et al., 2007), but it is not known if spontaneous activity occurs in vestibular HCs or neighboring SCs during postnatal development, or if calcium transients might play a role in maturation of vestibular sensitivity or neural circuits.

Here, we present direct evidence that spontaneous intracellular calcium transients [Ca^2+^]_i_ are present in vestibular HCs, SCs, and neuronal terminals in the first postnatal week of the developing mouse utricle. The genetically encoded reporter GCaMP5G was used to monitor calcium activity, and hence results reflect events detectable by this calcium indicator. Spontaneous calcium transients were present at birth in HCs, followed by spontaneous calcium transients in SCs and subsequently neuronal terminals. We further demonstrate that [Ca^2+^]_i_ transients in the utricular macula could be modulated by the efferent neurotransmitter ACh, as soon as postnatal day 3.

## Materials and Methods

### Animals and Strains

All animal experiments were approved by the Institutional Animal Care and Use Committee (IACUC) and conducted at the University of Utah in accordance with NIH guidelines. In the present work we used the wild-type strain C57BL/6J and two transgenic mouse lines: a dual Cre-dependent reporter Polr2a-based GCaMP5G-IRES-tdTomato, referred to as PC::G5-tdT (Gee et al., 2014) and a Gad2-IRES-Cre knock-in mouse driver line referred to as Gad2::Cre (Taniguchi et al., 2011). Both breeding pairs were obtained from The Jackson Laboratory [Polr2a^tm1(CAG−GCaMP5g, −tdTomato)Tvrd^ http://jaxmice.jax.org/strain/024477; Gad2^tm2(cre)Zjh^ http://jaxmice.jax.org/strain/010802]. First generation heterozygous offspring were used in these studies and hereafter referred to as Gad2-G5-tdT. Pups were genotyped using real time PCR (probes: “Polr2a-3”, “GCamp3-1 Tg”, and “tdRFP”; Transnetyx, Inc., Cordova, TN).

### Histological Preparation and Fixed Tissue Imaging

Mice of either sex at early postnatal ages (P1-P15) and adult controls (P73-P533) were used in this study. For immunohistological preparations, membranous labyrinths were harvested and immersion fixed in 4% paraformaldehyde overnight at 4°C. Whole mount utricle neuroepithelia were micro dissected, including the removal of otoconia, using fine forceps in 0.1 M phosphate-buffered saline (PBS) (Dumont #5, #55; Leica, M165 FC).

#### Whole Mount Preparation

Membranous labyrinths were incubated transiently three times in 0.1M PBS to remove residual aldehydes, followed by incubating in a blocking/permeabilization buffer [10% normal serum specific to the antisera (i.e., donkey or goat), 1% BSA, 0.5% Triton X-100 in 0.1M PBS] for 1-2 h on a nutating rocker platform at room temperature (RT). Tissues were subsequently incubated overnight at 4°C with a combination of the following primary antisera: anti-Atoh1 (SAB2100177, Sigma); anti-SOX2 (sc-365823, Santa Cruz); anti-Myosin-VIIa (25-6790, Proteus Biosciences). A monoclonal mouse anti-Tubulin β3 antibody conjugated with AlexaFluor® 647 was used at a dilution of 1:500 and included during primary antisera incubation (BioLegend cat# 801209). Tissues were subsequently rinsed three times for 15 min in 0.1M PBS, then placed in fresh blocking buffer and incubated for another 1 h at RT with secondary antisera conjugated to either AlexaFluor® [donkey anti-rabbit IgG H&L 405 nm (ab175651), DyLight™ [405 nm (cat# 35550), or 488 nm (cat# 35552)], Thermo Scientific, Inc.]. After incubations with secondary antisera, tissues were gently rinsed three times in PBS, placed onto glass bottom dishes (MatTek) submerged in PBS for confocal imaging (Olympus, FV1000). Z-stacks were acquired using a 5X air (N.A. 0.1), 40X water (N.A. 0.8), or 60X water (N.A. 1.0) objective with a digital zoom up to 3, and aspect ratio of 620 x 620 pixels.

#### Cryostat Sectioned Preparation

Intact bony labyrinths from P15 mice were harvested, and immersion fixed in 4% paraformaldehyde overnight at 4°C. Fixed intact labyrinths were placed in 5% EDTA in 0.1M PBS at RT and checked daily for decalcification and softening (up to 5 days). Once the bony labyrinths were pliable they were removed from EDTA, rinsed in fresh 0.1M PBS, and placed in a solution of 30% sucrose in 0.1M PBS to cryo-protect tissue. When tissues were no longer buoyant in the sucrose solution they were placed in a 1:1 solution of 30% sucrose: OCT for infiltration for ~1 h. Each labyrinth was placed in a mold containing OCT and oriented so that the oval window was facing up and basilar membrane was perpendicular to the bottom surface of the mold. Molds were frozen on dry ice and wrapped in parafilm to prevent evaporation until sectioning (Leica CM3050s; Prof Altschuler's laboratory, University of Michigan). Sections of 10–15 μm were collected on electrostatically charged slides (Superfrost™ Plus Slides, Fisher). Slides were kept at −20°C until processed for immunohistochemistry. Regions of tissues were optically examined and areas of interest were circled using a hydrophobic pen (Z377821, Sigma) and allowed to dry. Tissues were rehydrated in 0.1M PBS for 20 min and permeabilized in 0.1% Triton-X (PBS) for 20 min at RT. Permeabilization buffer was carefully removed and primary antisera (as described in the whole mount preparation section above) were added in fresh permeabilization buffer and incubated for 2 h at 37°C. Slides were rinsed in 0.1M PBS three times for 10 min with gentle rocking. Secondary antisera dilutions were added to tissues and incubated for 1 h at RT and subsequently rinsed in PBS as described for primary antisera. Finally mounting media was added and coverslips placed over tissues and allowed to dry at RT overnight. Images were obtained on Olympus FV1000 using a 60X oil immersion confocal microscope, images were post processed in FluoRender, and formatted for publication in Adobe Illustrator.

### Live Tissue Imaging and ACh Stimulation

Membranous labyrinths were micro-dissected from Gad2-G5-tdT temporal bones (P1-P15; and adults up to age P533), in cold glycerol-modified Ringer's (in mM: 26 NaHCO_3_, 11 glucose, 250 glycerol, 2.5 KCl, 1.2 NaH_2_PO_4_, 1.2 MgCl_2_ and 2.4 CaCl_2;_ pH 7.4) (Rabbitt et al., 2016). The utricle, horizontal and anterior cristae were further dissected to expose the apical surfaces of sensory neuroepithelia (Lim et al., 2011). Tissues were transferred to an imaging chamber (Warner Instruments; RC-22C) and continuously perfused with buffer (5.8 KCl, 144 NaCl, 0.9 MgCl_2_, 1.3 CaCl_2_, 0.7 NaH_2_PO_4_, 5.6 glucose, 10 HEPES, 300 mOsm, pH 7.4) at 21°C (Sadeghi et al., 2014). The recording chamber was placed under an upright swept field confocal microscope (Bruker; Prairie SFC), and imaged with a water immersion 60x objective (Olympus, LUMPLFN60XW). Confocal images were collected using a 35 μm slit aperture in linear galvanometer mode, and a 512 × 512 detector (Photometrics, Rolera MGi Plus EMCCD) providing in-plane single pixel size of 0.27 × 0.27 μm. For G5 detection and tdT expression imaging, 488 and 561 nm lasers, respectively, were interleaved for excitation, and a blocking filter (Semrock, R405/488/561/635-25) was used for detection. For G5 fluorescence calcium imaging, excitation was limited to 488 nm and the detection filter was replaced with a band pass filter (Semrock 525/50-25) to block any residual tdT emission at the 488 nm excitation wavelength. A pressure driven perfusion system configured with a micro-manifold (ALA Scientific Instruments, VC3-4PP, uflow-4) was used to continuously perfuse the tissue with control media, or 100 μM-1 mM ACh, 100 μM muscarine, 50 μM of the membrane permeable D-myo-inisitol trisphosphate receptor (IP_3_R) antagonist 2-aminoethoxydiphenyl diphenylborinate (2-APB, Sigma), or 5 μM muscarinic cholinergic receptor antagonist atropine (ATR). A computer controlled micro-manifold system was used to rapidly switch between media, ACh, 2-APB or ATR. The SFC confocal, stage position (Scientifica), and perfusion system (ALA, VM4) were controlled and monitored at 10 kHz by custom software (WaveMetrics, Igor) via AD hardware (Heka, ITC-18), IEEE-488 instrumentation interface (National Instruments, GPIB-USB; Sony/Tektronix, AFG-320) and serial interface (USB).

### Experimental Design and Statistical Analysis

G5 fluorescence was imaged at each “z” focal depth in a time sequence with images collected before, during, and after each ACh puff. Unless otherwise noted, image sequences consisted of 600–1,000 frames collected at 10 frames per second. The puff of each drug was delivered 10 s after initiating the sequence for a duration of 500 ms, unless otherwise noted. The image acquisition time sequence and ACh stimulus was repeated for each z focal depth (x,y,z) to generate 4D XYZT image stacks. Each xy image was smoothed in (x,y) space with a 3 pixel Gaussian filter (WaveMetrics, Igor Pro). To correct motion artifact, time-sequences at each focal plane were registered prior to analysis for full image rotation and translation (Thévenaz et al., 1998). G5 fluorescence modulation was determined pixel-by-pixel using ΔF/F_min_, where ΔF = F(t)–F_min_, and F_min_ was the minimum fluorescence intensity pixel-by-pixel over the entire time sequence of images. Image processing and pseudo-color images were generated in Igor Pro (WaveMetrics). Statistical significance of differences in the mean between groups was determined using Student's *t*-test with *p* = 0.05. All population data were presented as a mean ± standard error of the mean (SEM).

## Results

### Characterization of the Gad2-G5-tdT Transgenic Mouse Vestibular System

**Figure 1:**
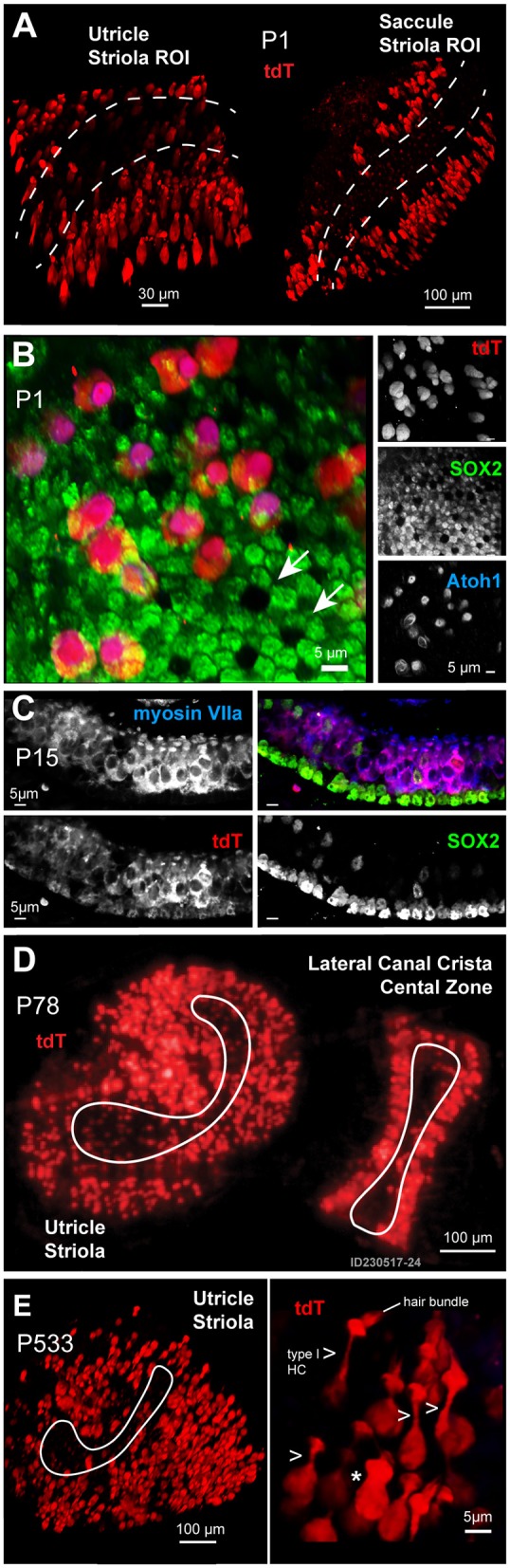
**(A)** Expression of the reporter tdT (red) was found primarily in a subset of extrastriolar cells in the utricle and saccule at the earliest time point examined, postnatal day 1 (P1). Images are maximum intensity projections (MIPs) from 70μm z-stacks through excised tissues. The striola in both the utricle and saccule are outlined with dotted lines. **(B)** Cells expressing tdT (red) at P1 immunolabeled with an antibody against ATOH1 (blue) and were identified as immature hair cells (HCs), while supporting cells (SCs) immunolabeled with an antibody against SOX2 (green). **(C)** By age P15, tdT (red) expressing cells immunolabeled with myosin VIIa (blue), with a few tdT negative hair cells immunolabeled with the SOX2 antibody (green). **(D,E)** Cells expressing tdT (red) had a flask-shaped morphology (arrows) and maintained their extrastriolar location within the utricle in adults (**D**: P78) and geriatric ages (**E**: P533).

At the earliest age examined in this study, postnatal day 1 (P1), a unique population of developing HCs and SCs express the transgene tdTomato (tdT) (Figure 1). These tdT expressing cells map to the extrastriolar regions of the otolithic organs in all first generation heterozygous animals. A maximum intensity projection (MIP) from confocal images taken through seventy-microns of the utricle and saccule (1 μm per slice) shows tdT expressing cells primarily in the extrastriolar region of the utricle and saccule in Gad2-G5-tdT mice at P1 (Figure 1A; Olympus FV100, 40XW obj). Immunolabeling was performed with the SC marker, SOX2, and the developmental HC differentiation marker, ATOH1, to further identify the tdT expressing cell types at P1 in the utricle (Figure 1B). One of the distinguishing morphological features of HCs and SCs during early postnatal developmental stages are their mosaics, which coincide with the Delta-Notch signaling (Figures 1B,C). Immunolabeling with SOX2 antibody revealed a mosaic pattern with six SCs surrounding a single HC (white arrows) by age P1 in the utricle (Figure 1B, green). During this early postnatal age, the tdT expressing cells immunolabeled with ATOH1 (Figure 1B, blue), but not SOX2, suggesting that at P1 these cells constitute a more sensory hair cell function (Figure 1B, blue). By postnatal day 15 (P15), tdT expressing cells (Figure 1C, red) remained predominantly sensory HCs (Figure 1C, myosin VIIa blue). SCs immunolabeled with SOX2 also showed tdT fluorescence, but at a low level relative to HCs (Figure 1C). The extrastriolar HCs and SCs expressing tdT by the end of the first two postnatal weeks persisted throughout adulthood. This extrastriolar tdT cell population is illustrated in a low magnification (SFC, 10X air obj) image of live tissue excised from an adult mouse (Figure 1D, P78) (Figure 1D). At the oldest age examined in the present study, postnatal day 533 (P533), tdT expressing cells maintained their extrastriolar location in the utricle. A representative image fixed whole mount utricle is shown from 110 μm confocal z-stack MIP where an ROI is centered on the utricle striola (white outline; Figure 1E Olympus FV1000, 40XW obj). Cells with prevalent tdT expression have hair bundles, although not all hair bundles are visible in the surface preparation shown. Two main cell morphologies among these tdT expressing cells were observed in mature tissue; cells with a long neck and hair bundles typical of type extrastriolar I HCs (arrows; 7 cells in ROI shown; Figure 1E, right panel); and a small number of cells lacking the characteristic long neck. This small subset of cells was not positively identified in the present study, and could be atypical shaped type I HCs, type II HCs, or other cell types.

### Spontaneous Calcium Transients in Hair Cells at Birth

**Figure 2:**
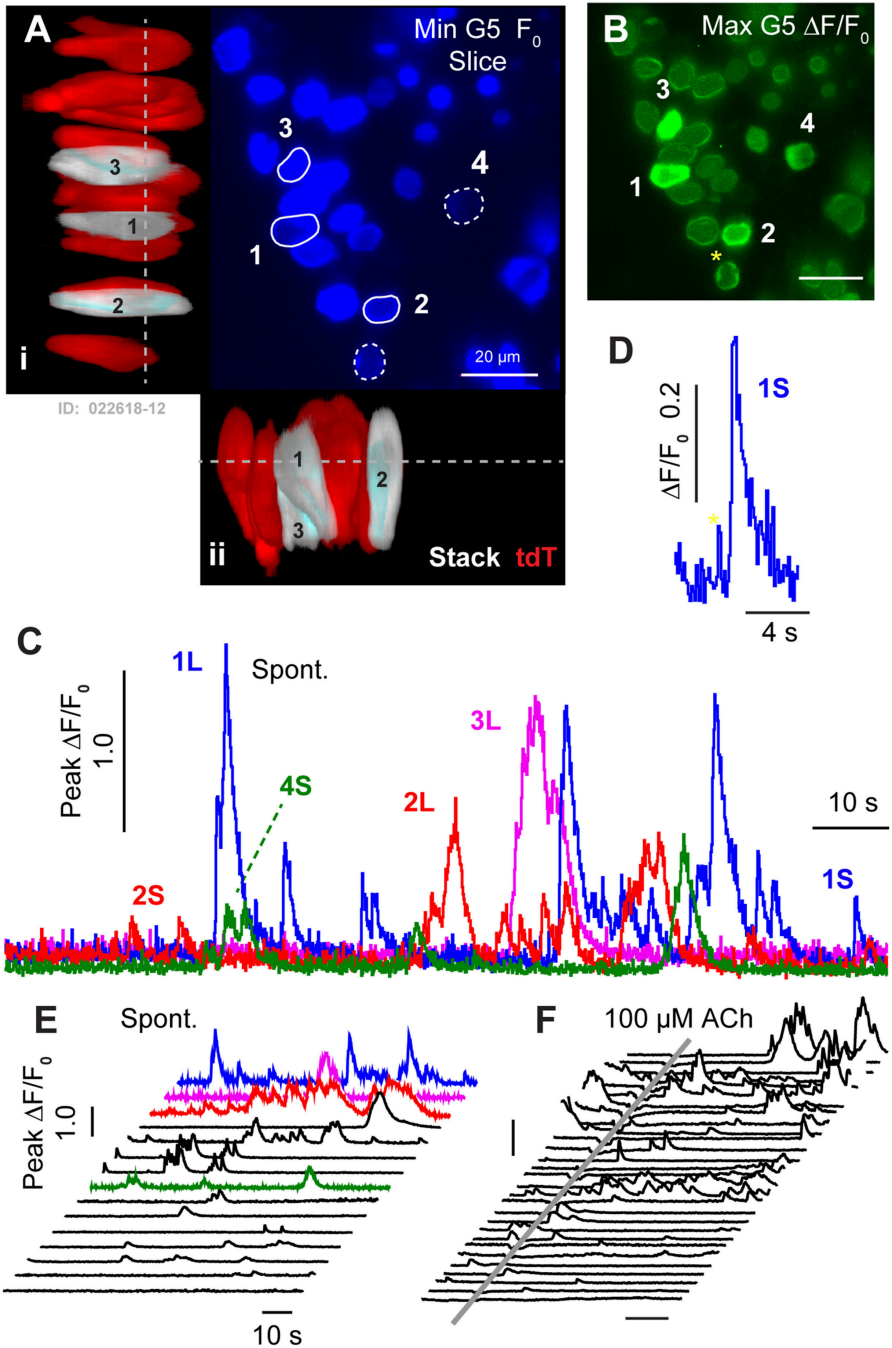
Spontaneous [Ca^2+^]_i_ transients in utricular HCs at P1. **(A)** Resting G5 fluorescence in HCs (F_0_, blue) in a plan view optical slice, and orthographic projections through the thickness of the epithelium showing expression of tdT (red & white) in HCs. Nominal Z-focus of the plan view is indicated with dashed lines. Three highlighted HCs exhibiting ΔF/F_0_ transients are pseudo-colored white (1–3). ROIs surrounding 5 cells are shown (white curves, 1–5). **(B)** Spontaneous [Ca^2+^]_i_ transients (peak G5 ΔF/F_0_ over 100 s, green) in tdT expressing HCs, sampled at ten frames per second. SCs lacking resting G5 fluorescence did not exhibit detectable spontaneous transients at P1. The largest [Ca^2+^]_i_ transients in HCs were located near the plasma membrane (e.g., 4*). **(C)** ΔF/F_0_ transients were complex in temporal waveform, as if consisting of the superposition of **(D)** small “S” events and large “L” calcium transients. **(E)** Spontaneous ΔF/F_0_ transients in HCs arranged by increasing magnitude, with colors corresponding to cells in **(A–C)**. **(F)** Application of ACh did not increase the rate or size of ΔF/F_0_ transients (arranged by increasing of ΔF/F_0_). Time near ACh application was blocked by optical interference from the wash. See Supplement Video 1.

**Figure 3:**
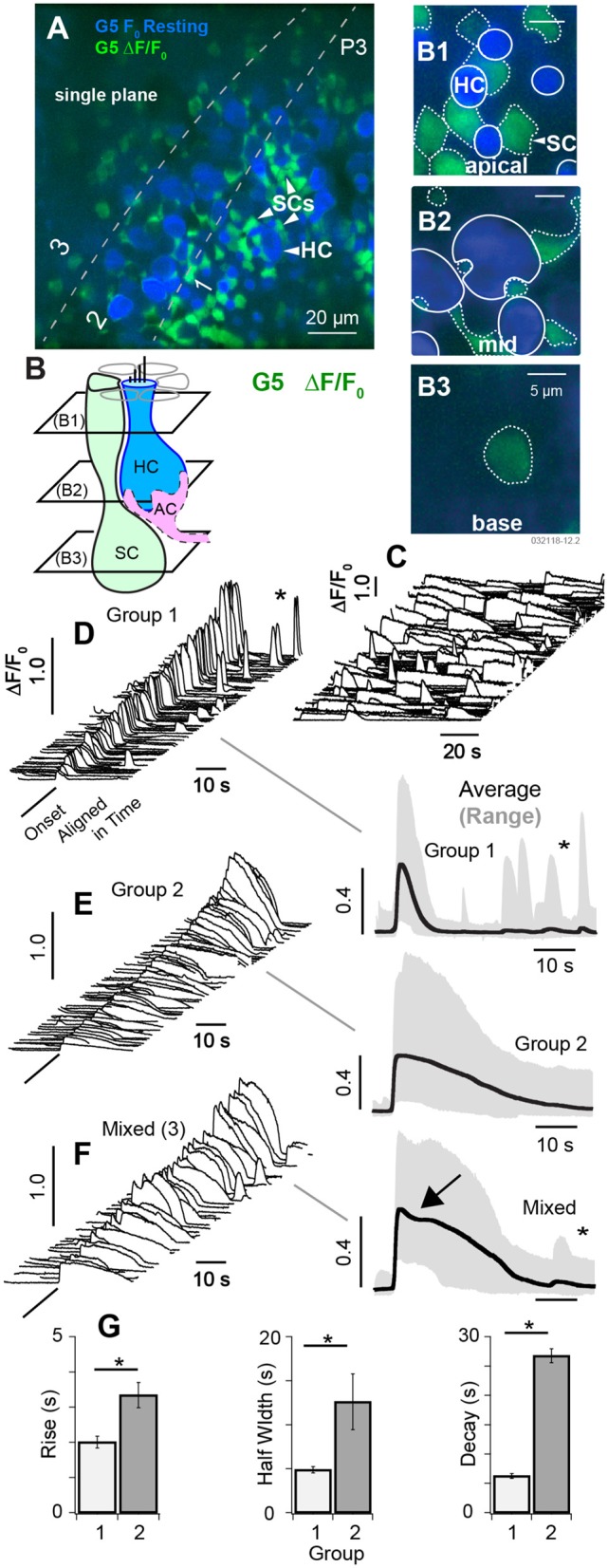
Spontaneous [Ca^2+^]_i_ transients in SCs at P3. **(A)** Single focal plane optically cutting through supporting cells (SCs) and hair cells (HCs) at 3 depths in the tissue (1: apical region, 2: central region, 3: below HCs). Resting, G5 fluorescence (F_0_ blue) and G5 ΔF/F_0_ transients (green) are superimposed. **(B)** Cartoon depicting 3 optical slices through SCs **(B1–B3)** and two through HCs **(B1,B2)**. The apical region of SCs and HCs **(B1)**, the middle region of SCs and HCs **(B2)**, and the base of SCs **(B3)**. Large spontaneous ΔF/F_0_ [Ca^2+^]_i_ transients were present in SCs, most easily identified by their hexagonal pattering around the apical necks of HCs **(A,B1)**. Solid white outlines show HC ROIs with large resting fluorescence, and dotted white lines show ROIs with large ΔF/F_0_ transients. **(C)** Example spontaneous ΔF/F_0_ transients sampled at 10 frames per second unaligned in time. **(D–F)** Spontaneous ΔF/F_0_ transients aligned in time and sorted by duration, magnitude, and waveform. ΔF/F_0_ transient waveforms show the average within 5μm ROIs centered on individual cells. **(D)** Group 1 short [Ca^2+^]_i_ transients with duration <10 s. Traces on the right show population averages (black) and range (gray). **(E)** Group 2 transients with durations >10 s. **(F)** Mixed transients with some events showing an inflection point just after the peak ΔF/F_0_ (arrow). **(G)** Population statistics over all ΔF/F_0_ transients from P3 aged mice showing the rise time, half width, and decay time by group. See Supplement Videos 2–4. *p = 0.05.

Spontaneous G5 ΔF/F_0_ transients ([Ca^2+^]_i_ transients) were observed in tdT expressing HCs at P1 (*n* = 166 cells analyzed; k = 3 P1 mice; see Supplement Video 1). G5 transients were imaged in excised utricles, with the confocal focal plane imaging transversely through the epithelium (Figures 2A–D). The minimum G5 fluorescence over all images in the time sequence was used to define the pixel-by-pixel *resting* G5 fluorescence, shown within one imaging plane (F_0_: Figure 2A; blue). After recording time sequences of G5 fluorescence modulation, tdT was imaged with 60 confocal planes to reconstruct reporter-expressing cells in Figure 3D. Cells expressing tdT in Figure 2A were identified as immature HCs based upon their morphology and immunolabeling (Figure 1). HCs are rendered as orthographic projections (Figure 2A i, ii) viewed perpendicular to the G5 confocal slice. HCs with high resting G5 fluorescence (Figure 2A, blue) also had abundant tdT expression (Figure 2A, red & white), indicating that resting G5 fluorescence in HCs primarily reflects expression of the reporter rather than high resting [Ca^2+^]_i_ in these cells. Three example HCs (1–3) exhibiting the largest spontaneous [Ca^2+^]_i_ transients in this tissue are rendered white to highlight their morphology and location (Figure 2A: i,ii). These same three HCs (Figure 2A: G5 F_0_, blue) are outlined in the orthogonal cross-section (Figure 2A i,ii) with solid white outlines (Figure 2F; 1–3) to define regions of interest (ROIs) for fluorescence modulation analysis. Two additional modulating HCs are outlined with white dashed outlines in one optical focal plane (Figure 2A; 4–5), but not rendered white in the 3D stacks.

**Figure 4:**
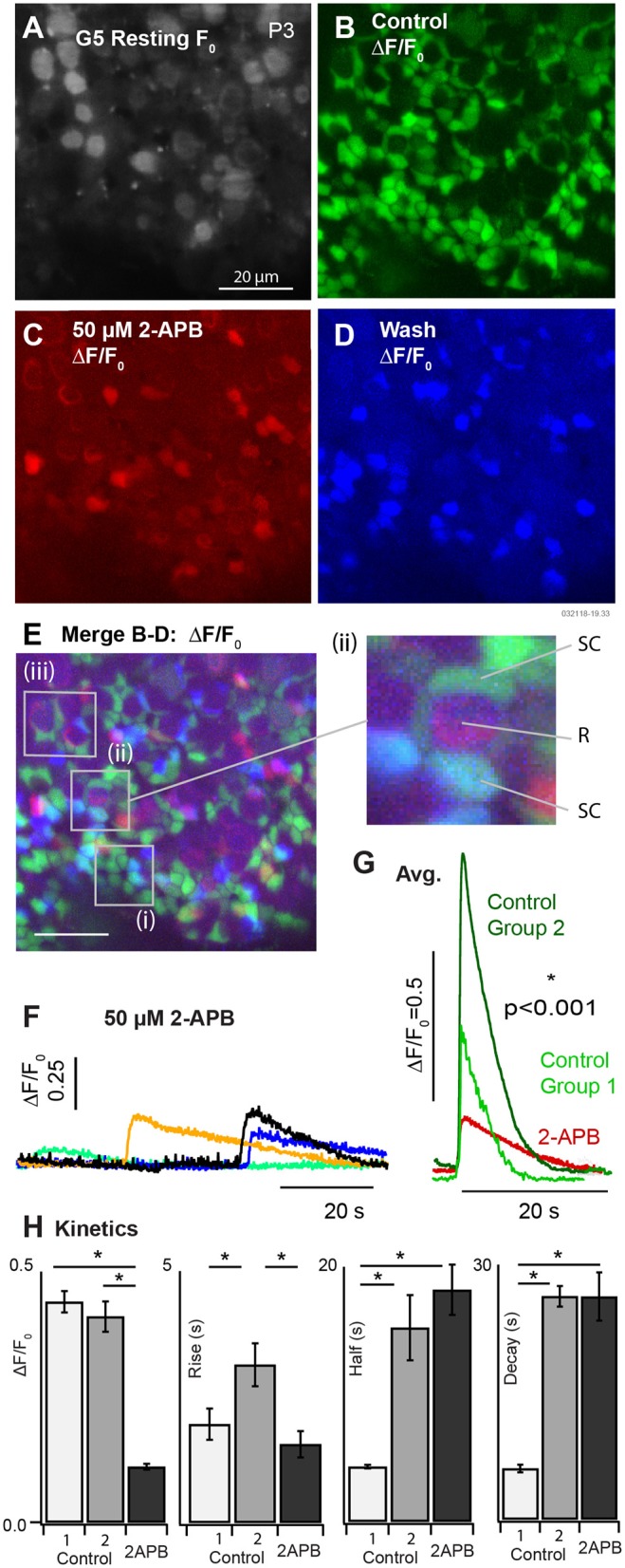
Partial block of utricular [Ca^2+^]_i_ transients by 50μM 2-APB at P3. **(A)** Resting G5 fluorescence, F_0_. **(B)** Spontaneous ΔF/F_0_ modulation (green) in the control condition. **(C)** ΔF/F_0_ modulation (red) after bath application of 50μM 2-APB. **(D)** Spontaneous ΔF/F_0_ modulation (blue) after washout of 2-APB. **(E)** Merge **(B–D)** showing modulation in the control condition (green), in the presence of 2-APB (red), and after washout (blue). The red channel was amplified to allow visualization of small ΔF/F_0_ in the presence of 2-APB. 50μM 2-APB reversibly blocked spontaneous activity in SCs. Cells that continued to respond in the presence of 2-APB were located near the base of HCs at P3. **(F)** Example transients in the presence of 2-APB, and **(G)** average waveforms in the control condition and in the presence of 2-APB. **(H)** Population statistics summarizing the action of 2-APB on the exponential rise time, half width, and decay time of ΔF/F_0_ transients. Spontaneous transients in the control condition are shown as fast events (Group 1) and slow events (Group 2) as in Figure 3. All events in the presence of 2-APB were slow and indistinguishable on the basis of kinetics.

[Ca^2+^]_i_ transients were tracked in time by computing ΔF/F_0_ pixel-by-pixel within outlined ROIs for each frame (1000 frames at 100 ms frame^−1^). Figure 2B. shows the maximum ΔF/F_0_ the over an example 100 s imaging sequence in this tissue. Of 35 HCs exhibiting resting G5 fluorescence in Figure 2 (F_0_ blue), an average of 5.4 (±1.46) cells per 138 × 138 μm^2^ focal plane exhibited spontaneous [Ca^2+^]_i_ transients (ΔF/F_0_ >0.5) within each 100 s image acquisition sequence. In the present report, we use the term “*spontaneous*” in reference to events in the baseline control condition, distinct from stimulus evoked modulation relative to the baseline condition. It is important to acknowledge that baseline spontaneous events observed in excised tissue depend on history and condition of the preparation, which differ from physiological conditions the cell may experiences *in vivo*. Spontaneous [Ca^2+^]_i_ transients in utricular HCs excised from P1 mice were not synchronized with each other, and occurred in different cells at different times (see Supplement Video 1). G5 ΔF/F_0_ transients were largest in magnitude near the plasma membrane, leading to a ring of increased ΔF/F_0_ near the membrane when imaged in cross section (Figure 2B; 1,2,4,5 examples). Although only 16% of 166 P1 HCs examined with prominent tdT expression exhibited spontaneous transients with ΔF/F_0_ > 0.5, 87% percent exhibited spontaneous transients at P1 with ΔF/F_0_ > 0.2 in at least one image during the sequence, indicating relatively rapid but small [Ca^2+^]_i_ fluctuations were likely present in the majority of utricular HCs expressing G5 at birth. [Ca^2+^]_i_ transients lasting only one frame were likely due to events shorter than the 100 ms exposure time and too fast to be resolved by the kinetics of the G5 fluorescent signal used in the present study (Podor et al., 2015). Temporal events with a half width < 1 s (10 frames) were not analyzed in the present report.

Spontaneous calcium transients are shown in Figure 2C for the 5 specific HCs highlighted in Figures 2A,B. Waveforms provide the peak ΔF/F_0_ within each outlined cell. Transients at birth consisted of small calcium increases, which we refer to as short “S” transients with characteristic waveforms (Figures 2C–F: 1S, 2S, 5S), large events with complex temporal waveforms (Figure 2C: 1L, 2L, 3L), and multiple medium size events falling between the two extremes. Other time sequences in this same tissue, and sequences from utricles excised from other P1 mice, also revealed 0–7 HCs with large and small spontaneous [Ca^2+^]_i_ transients within each 138 × 138 μm^2^ image plane. Additional spontaneous transients are shown in Figure 2E, arranged front to back by maximum ΔF/F_0_, with cells from panel C shown in the same colors. Spontaneous HC [Ca^2+^]_i_ transients rising above the background fluorescence fluctuation occurred with an average rate of 0.035 s^−1^ (±0.026) in P1 mice. Small events had a rise time to peak of 0.659 s (±0.062), a half width of 1.26 s (±0.075), and a decay time of 3.80 s (±0.297). Large transients “L” emerged from the background with a steep slope, consistent with the rise of S events, and had multiple peaks and jagged waveforms consistent with the hypothesis that large events might have resulted from the summation of multiple small S events occurring at different times within each HC. If large transients indeed occurred due to a superposition or burst of S events (Figures 2E,F, 3G), the rate of spontaneous Q events would be predicted to have occurred at an average rate of 2.19 s^−1^ (±2.74; range 0–4.6). [Ca^2+^]_i_ transients recorded in P1 HCs appeared to be “spontaneous”, and did not synchronize with each other in time. We were also unable to detect synchronization or modulation of HC [Ca^2+^]_i_ transients at P1 by ACh. This is illustrated by comparison of spontaneous transients (Figures 2E,F; independent experiments) to transients in after a 500 ms puff application of 100 μM ACh (Figure 2F) in the same tissue, where the time average ΔF/F_0_ was not changed by ACh. Data during ACh application was blocked due to image distortion caused by the fluid motion. Although, previous reports suggest METs can be functional as early as embryonic development (Géléoc and Holt, 2003), we did not detect fluid jet evoked G5 fluorescence modulation at P1, suggesting HC MET currents in this preparation were either not functional or insufficient to evoke [Ca^2+^]_i_ transients detectable by the G5 indicator at P1.

### Spontaneous Calcium Transients at P3

At postnatal day P3, spontaneous [Ca^2+^]_i_ transients observed in HCs were largely superseded by a period of spontaneous [Ca^2+^]_i_ activity in cells with low resting G5 fluorescence consisting primarily of SCs (see Supplement Videos 2, 3). Figure 3A shows a single confocal slice cutting through HCs, SCs, and immature afferent calyces (AC). Using the same color format as Figure 2, the minimum fluorescence over the 100 s time sequence of images was used to define the resting fluorescence (Figures 3A,B, min F_0_; blue), and the change in fluorescence (ΔF/F_0_, green) was used to track changes in [Ca^+2^]_i_, with Figures 3A,B showing the superposition of the two. The confocal optical slice was tilted relative to the epithelium in this tissue, cutting through the apical necks of hair cells in zone 1 (Figures 3A,B1), basolateral region in zone 2 (Figures 3A,B2), and below hair cells in zone 3 (Figures 3A,B3). Over 160 cells exhibited detectable G5 fluorescence in each 138 × 138 μm^2^ image and, of these, 97 (±19.6) cells per focal plane exhibited G5 spontaneous calcium transients (874 events per experiment). SCs were easily identified in apical regions of live tissue based on their hexagonal spatial patterning around HCs (Figure 3B, also see Figure 1). Solid white lines in Figure 3B1 show HC ROIs with high resting G5 fluorescence (blue) while dotted white lines show SC ROIs with relatively low resting G5 fluorescence, but high ΔF/F_0_ transients (green). Type I HCs were identified based on their relatively high G5 resting fluorescence (blue) and tdT expression (e.g., Figure 1), as well as their characteristic morphology. Although ΔF/F_0_ transients were observed near the plasma membrane of some HCs, these events did not extend into HC somata. The spatial resolution was insufficient in the present imaging paradigm to determine if these localized events were in HCs or adjacent cells, Spontaneous G5 ΔF/F_0_ transients were present at all focal depths, including below HCs. Although spontaneous G5 transients in the apical zone (A1,B1) were clearly localized in SCs at P3, SCs could not be positively distinguished from other key cell types at deeper focal planes, leaving open the possibility that some spontaneous transients might also be present in afferent neurons or calyces (ACs) at P3 as well.

In utricles excised from P3 mice, spontaneous transients were not synchronized with each other and occurred in different cells at different times (Figure 3C, Supplement Videos 2, 3). Transients were divided into 2 groups for statistical analysis based on kinetics of the G5 ΔF/F_0_ temporal waveforms. Group 1 transients were distinguished by their relatively short duration (< 10s) relative to Group 2. A third set of ROIs (3) had mixed [Ca^+2^]_i_ transients characterized by an inflection point (Figure 3E, arrow), and possibly arising from fluorescence events from ΔF/F_0_ reporting a combination of two events with different waveforms occurring within the same ROI or from distinct calcium kinetics (Taheri et al., 2017). Example events are shown in Figures 3D–F (left), aligned in time and arranged in increasing ΔF/F_0_ magnitude. Population averages are shown on the right, and population statistics are summarized in Figure 3G. Short duration Group 1 transients (Figure 3C) had an average rise time of 1.96 s, half width of 4.31 s, decay of 6.00 s and peak ΔF/F_0_ of 0.44 (data not shown). Long duration Group 2 transients had an average rise time of 2.76 s, half width of 11. 7 s, decay of 25.3 s and peak ΔF/F_0_ of 0.40 (Figure 3G). Mixed (3) transients had an average rise time of 2.98 s, half width of 18.8 s, decay of 27.2 s (Figure 3G); and peak ΔF/F_0_ of 0.42 consistent with a superposition of events from groups 1 and 2 (data not shown). Transients in each group occurred with similar ΔF/F_0_ magnitude and could not be distinguished from each other based on ΔF/F_0_ magnitude alone. Occurrence of long transients (56%) modestly outnumbered short transients (44%). There was no detectable correlation between any group and focal depth in tissue at P3 (B1–B3 depths). Group 1 events were more likely to reoccur during the imaging, sequence leading to a second peak in time-domain traces (Figure 3C, ^*^). The average rate of spontaneous short events (Group 1) occurring within a single cell was 0.044 s^−1^ (±0.043) and long events within a single cell was 0.016 s^−1^ (±0.0035). Since ΔF/F_0_ transients at the apical surface were clearly identified as SCs, the lack of correlation with focal depth suggests ΔF/F_0_ transients might have been dominated by SCs at all focal depths through the epithelium in P3 aged mice (see Supplement Video 2). However, there were numerous exceptions at P3, based primarily on morphology, where spontaneous [Ca^2+^]_i_ transients likely occurred in other cell types including HCs, ACs, or other terminals at the base of HCs (see Supplement Videos 3,4).

We applied 50 μM 2-APB to block spontaneous [Ca^2+^]_i_ transients in SCs of P3-5 aged mice B (*k* = 3 mice). Example G5 F_0_ and ΔF/F_0_ are provided in Figure 4 in the control condition (A: resting F_0_, gray and B: ΔF/F_0_, green), blocked condition (C: ΔF/F_0_, red), and after wash (D: ΔF/F_0_, blue). G5 ΔF/F_0_ transients were reduced more than 5 fold after application of the IP_3_R antagonist 2-APB. G5 ΔF/F_0_ images were merged (Figure 4E) to reveal specific cells that lost spontaneous [Ca^2+^]_i_ modulation after application of 2-APB. A vast majority of SCs had a complete cessation of ΔF/F_0_ transients in the presence of the antagonist as evidenced by the near absence of overlap between the red and green fluorescent channels (Figure 4E, overlap = yellow). Although washout was incomplete, a subset of SCs clearly recovered spontaneous ΔF/F_0_ transients (Figure 4E, recovery = blue-green, e.g., insets i & ii). SCs are arranged in canonical patterns of 6 around the necks of HCs, corresponding to the locations of SC ΔF/F_0_ transients (Figure 4E, i). A subset of cells continued to show low-intensity spontaneous ΔF/F_0_ transients in the presence of 50μM 2-APB (Figures 4C,E, red). Although the precise identity of these cells is uncertain due to their developmental age and the likelihood that they are actively undergoing differentiation, modulating ΔF/F_0_ domains often localized with tdT positive HCs at their base (e.g., Figure 4E i & ii R), suggesting potential origins in other cell types, possibly HCs or developing ACs at this age. On average, the few [Ca^2+^]_i_ transients that remained in the presence of 50μM 2-APB were relatively small and slow compared with pre-treatment control conditions. Specific examples are shown in Figure 4F, with average transients for all cells in the control condition compared with 2-APB treated cells in Figure 4G. Averages in the control condition are shown for [Ca^2+^]_i_ transients lasting < 10 s (Group 1) and for transients lasting > than 10 s (Group 2). Population statistics comparing responses in the control condition vs. in the presence of 2-APB are provided in Figure 4H. Group 1 events in the control condition for mice tested with 2-APB (Figure 4, P3-P5) were slightly faster than Group 1 events at P3 (Figure 3), but this study did not determine if this was due differences in maturation or some other unidentified factor. Compared to Group 1 events in the control condition, [Ca^2+^]_i_ transients in the presence of 2-APB had a reduced peak ΔF/F_0_ (0.44±0.21 vs. 0.11±0.023), increased decay time (5.99 ± 0.45 vs. 26.26 ± 2.84 s), with no statistically significant difference in rise time. Compared to Group 2 events in the control condition, [Ca^2+^]_i_ transients in the presence of 2-APB had a reduced peak of ΔF/F_0_ (0.41 ± 0.24 vs. 0.11 ± 0.023), a reduced rise time (3.14 ± 0.43 vs. 1.56 ± 0.26 s), with no significant difference in half width or decay time. These results demonstrate that 2-APB interferes with spontaneous [Ca^2+^]_i_ transients, significantly reducing the magnitude of all events and reducing the number and kinetics of short duration [Ca^2+^]_i_ transients in multiple developing cell types. Antagonist action of 2-APB on IP_3_R is sufficient to explain these results through disruption of CICR. However, the present experiments did not completely rule out other hypothetical [Ca^2+^]_i_ mechanisms, which is the focus of future studies.

**Figure 5:**
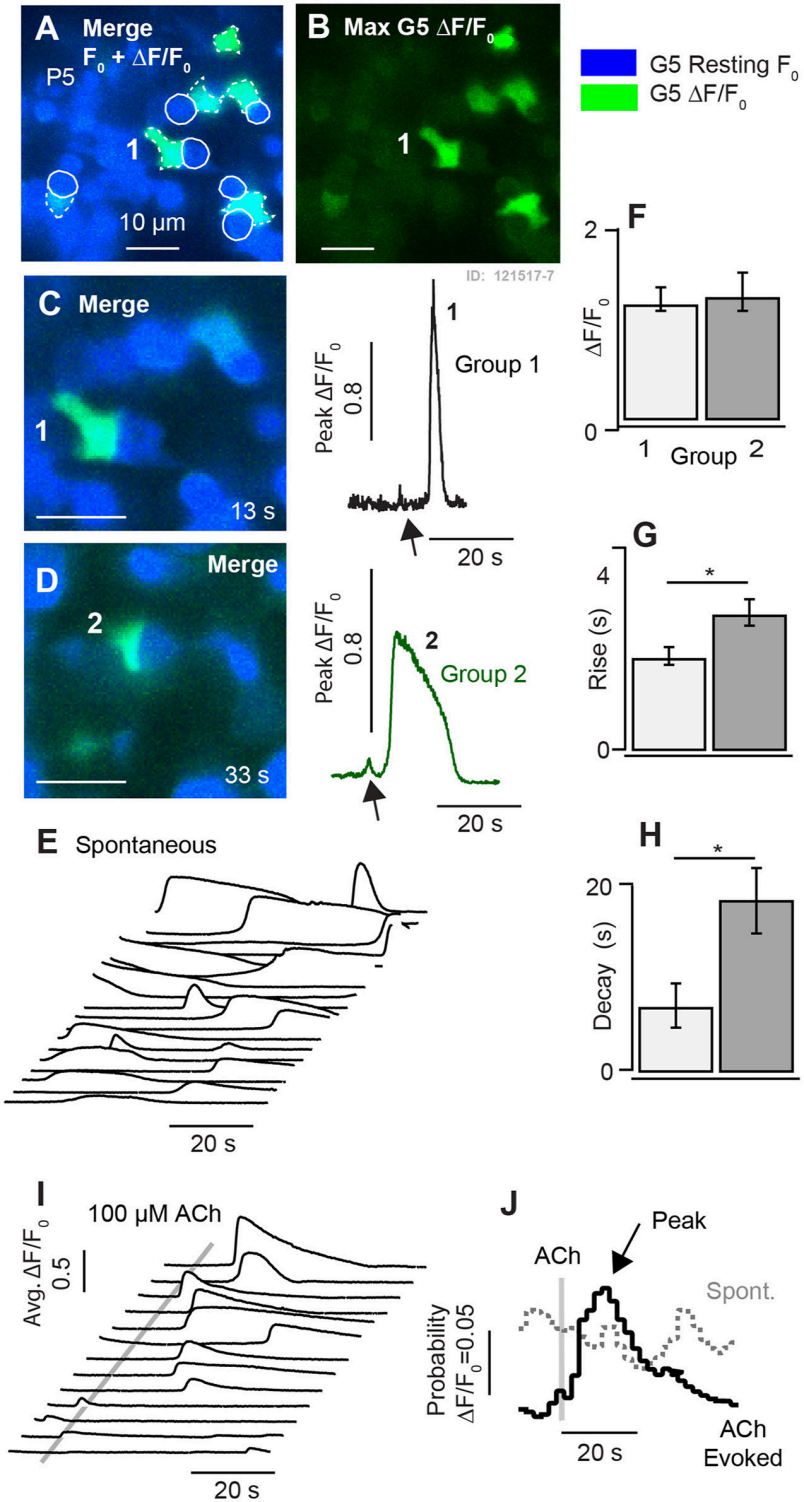
Synchronization of [Ca^2+^]_i_ transients by 100μM ACh at P5. **(A,B)** Resting G5 fluorescence (blue) and spontaneous [Ca^2+^]_i_ transients based on the maximum G5 ΔF/F (green) over a 100s time sequence imaged at 10 frames per second. **(C,D)** Two example instants in time showing spontaneous transients in example cells (1–2). **(E)** Time sequence of spontaneous ΔF/F_0_ transients in individual cells. **(F–H)** Population statistics at P5 showing the size, rise time, and decay time of fast (Group 1) and slow (Group 2) transients. **(I)** Time sequence showing synchronization of ΔF/F_0_ transients by puff application of 100μM ACh. **(J)** Stimulus averaged ΔF/F_0_ transients illustrating synchronization by ACh. *p = 0.05.

At postnatal day P5, ΔF/F_0_ [Ca^2+^]_i_ transients were similar to those observed in P3 mice (Figure 3), but significantly fewer in number, occurring in only 4.7 (±1.8) cells per 138 × 138 μm^2^ focal plane. Figure 5 provides example data recorded at a single focal plane (Figures 5A–J), and with population statistics across animals (F-H: *n* = 75 cells; *k* = 3 mice). Using the same approach as earlier ages, spontaneous [Ca^2+^]_i_ ΔF/F_0_ transients at P5 were grouped based on duration, with cells having ΔF/F_0_ duration < 10 s placed in Group 1 and others placed in Group 2 (Figures 5E–H). Similar to P3 aged mice, these two groups at P5 had similar ΔF/F_0_ peak magnitudes (Group 1 0.19 ± 0.04 vs. Group 2 0.20 ± 0.03; Figure 5F), but statistically significant differences in kinetics (Figures 5G–H). Compared to events in Group 1 transients in Group 2 had a slower rise time (1.32 ± 0.17 vs. 3.56 ± 0.49 s; Figure 5G), increased half width (4.59 ± 0.68 vs. 17.7 ± 1.75 s; data not shown), and longer decay times (8.88 ± 2.22 vs. 19.9 ± 1.79 s; Figure 5H). Smaller ΔF/F_0_ events approaching the kinetics of G5 resolution occurred in some cells from both groups (C1, D2, arrows). Although the small events sometimes appeared before the large [Ca^2+^]_i_ transients, most large transients were not preceded by detectable small events suggesting the small [Ca^2+^]_i_ transients are not likely to be required precursors. Both types of [Ca^2+^]_i_ transients occurred spontaneously in the control condition, at random times in the imaging sequence. This is illustrated in Figure 5E where individual traces recorded in one focal plane show ΔF/F_0_ transients from individual cells. Domains exhibiting spontaneous transients in utricles from P5 mice were often complex in morphology and contacted the basolateral membranes of HCs, similar to domains showing spontaneous transients near the base of HCs at earlier ages.

### Development of ACh Evoked [Ca^2+^]_i_ Transients

Although ACh application did not evoke G5 detectable [Ca^2+^]_i_ (ΔF/F_0_) transients in utricles from P1-P3 aged mice, we hypothesized that ACh would begin to evoke G5 detectable [Ca^2+^]_i_ transients as calyces developed due to the presence of muscarinic AChRs (Holt et al., 2017). In utricular maculae excised from P5 aged mice, puff application of 100 μM ACh indeed synchronized the timing of ΔF/F_0_ transients. Figure 5 compares spontaneous ΔF/F_0_ [Ca^2+^]_i_ transients (Figure 5E) to ACh synchronized transients (Figure 5I) in the same cells for repeated application of ACh. Traces were collected in time sequences, repeated following a 5s delay at 4 sequential focal planes. Cells became refractory immediately following a calcium transient, requiring a period of time to recover before a subsequent transient could be evoked by ACh or occurred spontaneously. This is consistent with similar calcium transients observed in astrocytes (Taheri et al., 2017), and is likely due to the long time course of CICR dynamics and signaling. The refractory time during repeated stimuli accounts for the lack of “spontaneous” activity prior to ACh application in Figure 5I (ACh stimuli were repeated in 45s intervals). Synchronization by ACh is illustrated in the form of average response histograms (Figure 5J) where spontaneous transients occurred at random times relative to the onset of the imaging sequence (dotted gray), but became synchronized by application of ACh resulting in a peak probability of a ΔF/F_0_ transient occurring at latency of 9.7 s (±7.1) after the ACh puff (Figure 5J, black).

**Figure 6:**
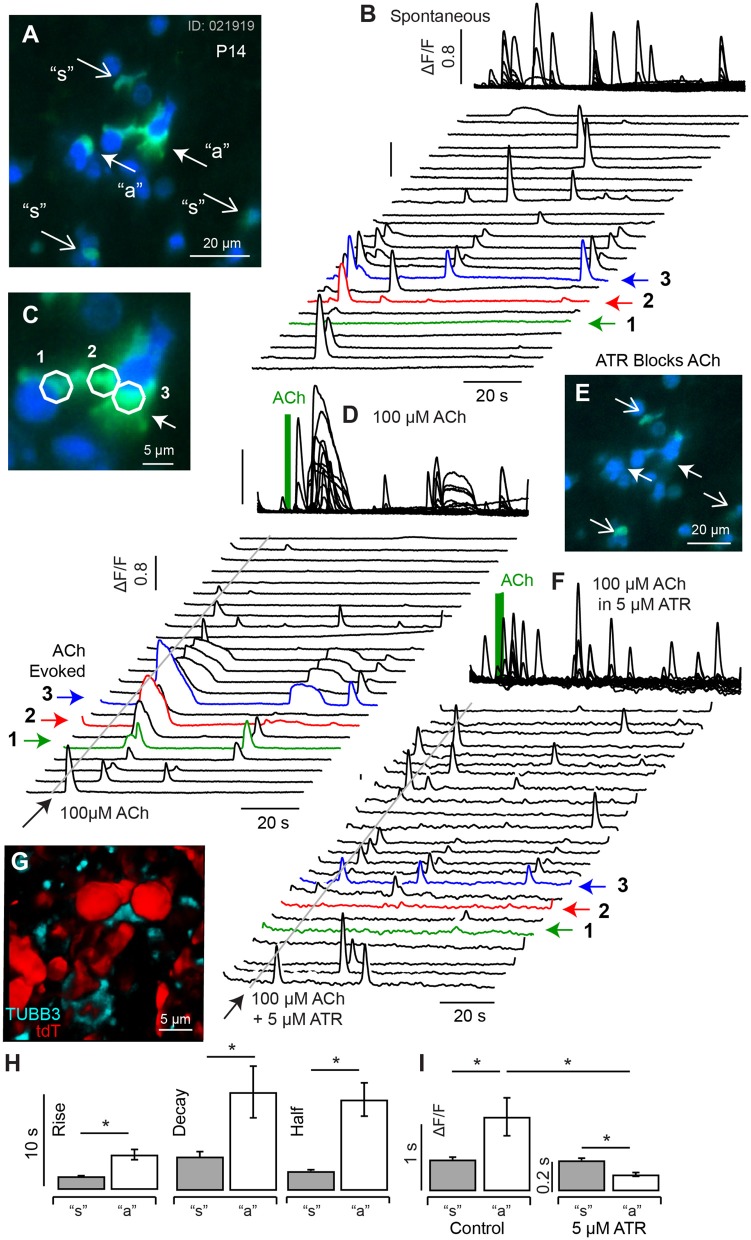
Spontaneous and ACh evoked [Ca^2+^]_i_ transients at P14. **(A)** Resting G5 fluorescence (blue) and peak G5 ΔF/F_0_ (green) showing superposition of spontaneous (“s” and ACh evoked events (“a”). **(B)** Spontaneous transients in 23 example 5 μm diameter ROIs, with transients in 3 ACh sensitive ROIs highlighted (1,2,3), at higher magnification of in **(C)**. **(D)** ACh evoked transients were observed in a subset of cells (e.g., 1,2,3), with morphology consistent with calyx terminals contacting hair cells. **(E)** G5 ΔF/F_0_ was blocked by 5μM ATR in a subset of cells (closed arrows) but persisted in other cells (open arrows). **(F)** ATR blocked long-lasting responses in ACh sensitive cells but not in other cells. **(G)** Confocal image of P14 fixed tissue showing the morphology of tdT positive hair cells (red) and synaptic contacts (anti-Tubulin) in an imaging slice similar to **(A)**, suggesting ATR-sensitive cells are calyx endings. **(H)** ACh evoked transients could be distinguished from spontaneous transients by their kinetics, with ACh evoked events rising more slowly and lasting longer. **(I)** ACh evoked transients were largely blocked by ATR, while short-duration transients were not. *p = 0.05.

ACh evoked transients became more pronounced in P10-14 mice. Figure 6 shows resting G5 fluorescence (A, blue), superimposed on peak G5 (ΔF/F_0_) (C, green) recorded over an 80 s imaging sequence (100 ms frame^−1^). For quantification, ΔF/F_0_ [Ca^2+^]_i_ transients were recorded in 5 μm diameter ROIs in cells with complex morphological shapes (Figure 6A: “a” closed arrows. Figures 6B,C: 1-red, 2-green, 3-blue) and in simple morphological shapes (Figure 6A: “s” open arrows, Figure 6B: black). Spontaneous transients exhibited similar kinetics in all ROIs and, on average, had a rise time 1.28 s (±0.045), decay time of 1.98 s (±0.083), and half width 1.59 s (±0.075) (Figures 6B,H). A puff application of 100 μM ACh for 1s evoked distinctly longer lasting ΔF/F_0_ transients in a subset of ROIs (Figure 6A: “a”. Figures 6C,D: 1-red, 2-green, 3-blue) which, on average, had a rise time 2.80 s (±1.45), decay time of 3.97 s (±1.60), and half width 3.37 s (±0.44). Population kinetics of ΔF/F_0_ [Ca^2+^]_i_ transients in ATR insensitive cells (“s”) vs. ATR-sensitive cells are summarized in Figures 6H,I. The long lasting ACh-evoked transients were blocked by the muscarinic ACh receptor (mAChR) antagonist ATR (5 μM, Figures 6E,F), with residual small transients persisting in some ACh sensitive ROIs (Figure 6F, 1-3). Cells responding to ACh with long lasting calcium transients had morphological shapes consistent with calyceal afferent endings contacting type I hair cells. This is illustrated by comparing the morphology of tubulin labeled afferent endings (cyan) contacting tdT hair cells (red) from the same P14 tissue after fixation (Figure 6G, maximum intensity projection from a 62 μm z-stack). Present data suggest ACh activation of mAChRs on calyceal endings triggers large and long lasting CICR by P14 (Figure 6) that likely plays an important role in responses of mature calyces to ACh and efferent activation (Holt et al., 2017).

**Figure 7:**
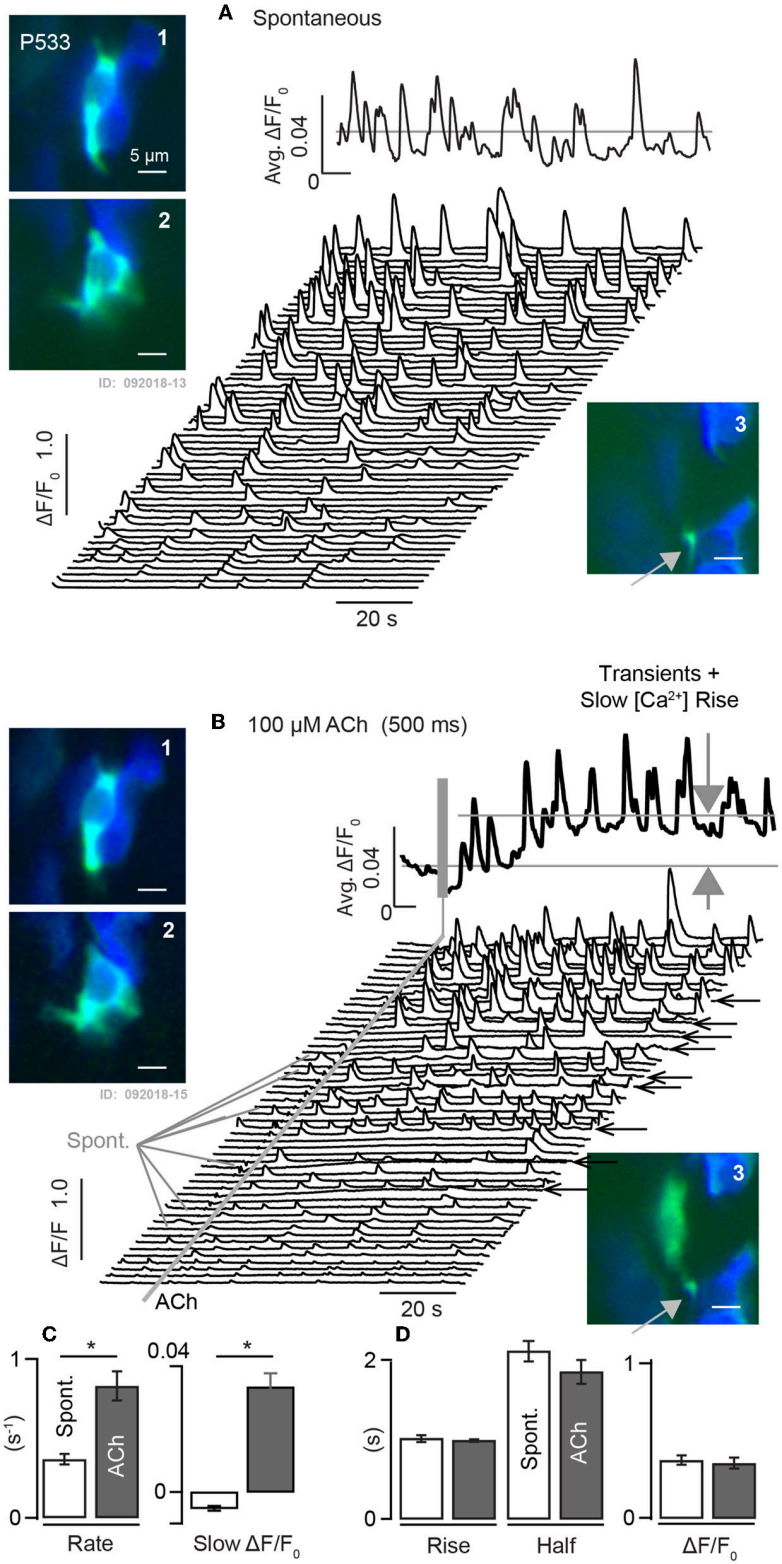
Spontaneous and ACh evoked [Ca^2+^]_i_ transients in utricular maculae from mature mice. **(A)** Spontaneous G5 ΔF/F_0_ recorded at 100ms-frame^−1^ in the control condition. Waterfall plot shows transients within 3μm diameter ROIs. Solid curve above the waterfall shows the average over all ROIs. A1-2: examples of ROIs within cells of calyceal morphology near the base of HCs, and A3: example with bouton morphology. **(B)** Same ROIs modulating in response to 1s puff application of 100μM ACh. ACh increased the rate of transient events and evoked a slow rise in [Ca^2+^]_i_ (large gray arrows). B1-3: same ROIs as **(A)**, but with transients evoked by ACh. B3: ACh extended the spatial extent of the transient relative to spontaneous events. Solid curve above the waterfall shows the average over all ROIs. **(C,D)** Population statistics demonstrating that ACh evoked a significant increase in the rate of fast transients as well as a slow increase in [Ca^2+^]_i_. No change was detectable in kinetics, duration, or magnitude of fast transients in the ACh condition relative to controls. *p = 0.05.

Intracellular calcium transients were also observed using G5 in utricles excised from adult mice (Figure 7). [Ca^2+^]_i_ transients recorded from a mature P533 aged mouse shows persistence of spontaneous ΔF/F_0_ transients in control conditions recorded in 18 distinct cells in this tissue at a sampling rate of 100 ms-frame^−1^. The average of these traces is shown as the solid black curve above the waterfall, and example cells are shown in inset images 1–3 (ΔF/F_0_.: green, F_0_ blue). Based strictly on morphology and contacts on HCs (blue) these modulating domains are likely to include calyceal endings near the base of HCs (1–2) and bouton terminals (3). The 18 cells in this tissue exhibited spontaneous transients at a rate of 0.37 s^−1^ (±0.033). When the same cells were exposed to 100 μM ACh for 1s the rate of transients increased to 0.83 s^−1^ (±0.091), (Figures 7B–D). ACh also evoked a slow increase in [Ca^2+^]_i_ evidenced by the slow increase of ΔF/F_0_ not present in the control condition (Figure 7B: large gray arrows, black trace). In some cells ACh extended the spatial extent of the intracellular [Ca^2+^]_i_ transient relative to spontaneous events in the same cell in the control condition (Figure 7: A-3 vs. B-3), possibly due to the activation of CICR these cells. Population statistics in this aged utricle confirms that ACh evokes a significant increase in the rate of fast transients in addition to a slow increase in [Ca^2+^]_i_ (Figure 7C), but there was no detectable influence on kinetics of the fast [Ca^2+^]_i_ transients as measured by the rise time, half width, or ΔF/F_0_ relative to controls (Figure 7D).

## Discussion

**Figure 8:**
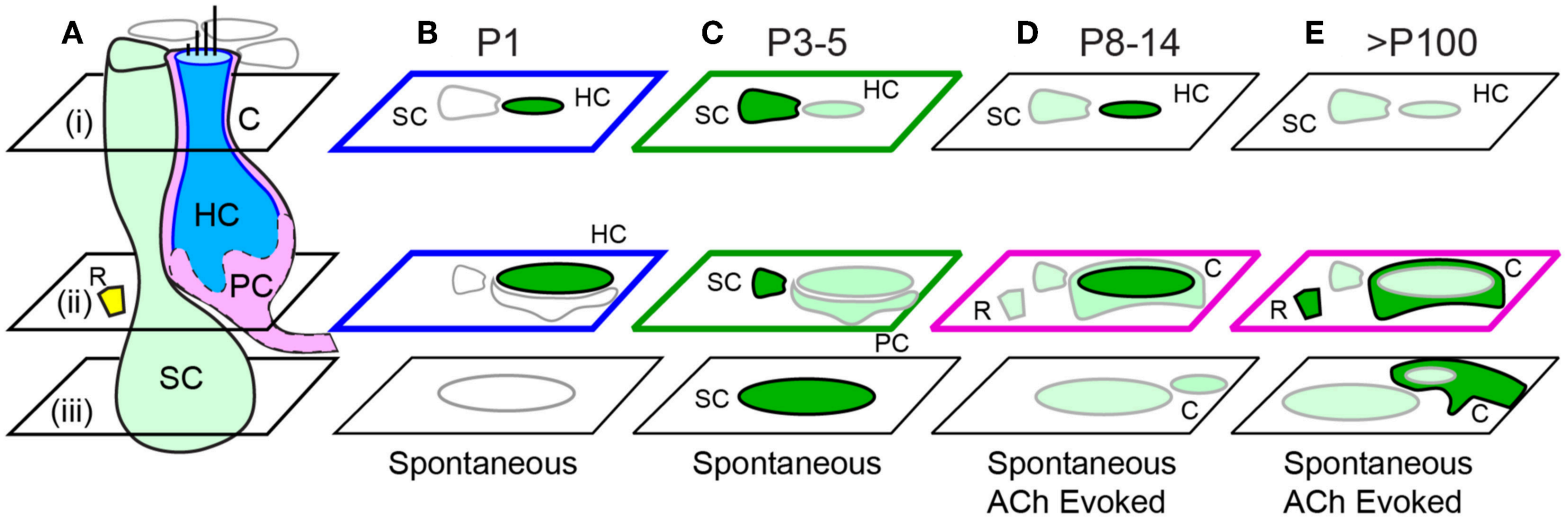
Summary of spontaneous and ACh evoked [Ca^2+^]_i_ transients with age. **(A)** Cartoon depicting a supporting cell (SC), extrastriolar type I tdT positive hair cell (HC), calyx ending **(C)**, immature proto-calyx ending (PC), and small ACh activated domains (R) near the base of HCs. Focal planes are denoted i-iii. **(B)** Intense spontaneous ΔF/F_0_ transients in immature HCs at P1. **(C)** Intense spontaneous ΔF/F_0_ transients in SCs and additional transients in HCs and PCs at P3-5. ACh synchronized of transients at P5. **(D)** Sparse spontaneous transients in SCs, intense ACh evoked transients in some HCs, and additional transients in Cs and Rs in P8 and older mice. **(E)** Spontaneous transients in SCs and intense ACh-evoked transients C endings.

Intracellular calcium transients during the first week of postnatal development in mouse utricular macula are summarized schematically in Figure 8. Spontaneous whole-cell [Ca^2+^]_i_ transients were present in HCs with tdT expression at birth (Figure 7B; also see Supplement Video 1) and were largely reduced in HCs at P3 (Figure 7C; Supplement Video 2). In mature mice from this transgenic cross, HCs with strong tdT expression are primarily extrastriolar type 1, which are known to be contacted by calyx endings of dimorphic afferent neurons (Eatock et al., 1998; Goldberg, 2012). If the tdT expression is maintained through postnatal development, HCs expressing tdT at P1 would be expected to have extrastriolar type I fate. Because of this, spontaneous HC transients reported here at P1 would be expected to drive action potentials in dimorphic afferents prior to the onset of mature vestibular function. Vestibular afferent neurons have irregularly spaced inter-spike-intervals at P1 (Desmadryl et al., 1986), consistent with the firing pattern expected in neurons that contact type I HCs (Rüsch et al., 1998; Goldberg, 2012), and consistent with spontaneous [Ca^2+^]_i_ transients observed in putative type I HCs examined here at P1 (Figures 1, 2). Present results support the hypothesis that extrastriolar type I hair cells generate spontaneous calcium activity at P1, which is likely to drive spontaneous action potential modulation in immature afferents with dimorphic fate. Since the G5 reporter was preferentially expressed in these specific HCs, present results cannot address possible spontaneous activity in other hair cell types or partner afferent neurons. Based on the developmental time course in humans, this same process might be expected to occur at 10–15 weeks gestation (Lim et al., 2014; Lim and Brichta, 2016).

Whole-cell spontaneous [Ca^2+^]_i_ transients in HCs became rare by P3, demonstrating a change in operation of these HCs (Figure 7C). The activity of utricular ganglion neurons also changes by P3, with some neurons beginning to discharge with regularly spaced inter-spike intervals, supplementing neurons discharging with irregularly spaced intervals (Desmadryl et al., 1986). The appearance of units with regular discharge statistics suggests HCs with type II fate are likely releasing neurotransmitter at P3 in addition to HCs with type I fate, although not examined directly in the present study. Spontaneous action potentials in vestibular ganglion neurons reported previously combined with the near absence of whole-cell ΔF/F_0_ transients in HCs reported here in P3 mice, suggests spontaneous neurotransmitter release from HCs at P3 does not require large whole cell [Ca^2+^]_i_ transients, but instead likely relies on smaller localized [Ca^2+^]_i_ transients similar to mature HCs, unlikely to be detectable by the present G5 indicator.

The pattern of spontaneous [Ca^2+^]_i_ activity changed dramatically by P3 when ubiquitous ΔF/F_0_ [Ca^2+^]_i_ transients appeared in utricular SCs (Figures 3, 4; Supplement Videos 2, 3). SC [Ca^2+^]_i_ transients occurred in scattered patterns across the epithelium (Figure 3; Supplement Video 2) and did not exhibit detectable intercellular calcium wave propagation from one cell to adjacent cells as might be expected from gap-junction communication. This appears to differ from the developing cochlea, where spontaneous calcium activity in Köllikers organ is propagated between adjacent cells and likely coordinates local purinergic signaling to inner HCs during early postnatal development (Tritsch et al., 2010; Zhu and Zhao, 2012; Dayaratne et al., 2014, 2015). Intracellular calcium transients in utricular SCs were likely IP_3_ dependent (Figure 4), but we did not observe [Ca^2+^]_i_ propagation that would be expected if connexin-dependent intercellular signaling between adjacent SCs was present at levels similar to the cochlea (Beltramello et al., 2005; Zhang et al., 2005). Although sequence variants in *connexin* genes are responsible for the most common forms of hereditary deafness (Cohen-Salmon et al., 2002, 2004; Chang et al., 2003; Teubner et al., 2003; Leibovici et al., 2008; Wan et al., 2013), vestibular function is not as profoundly impaired with these genetic mutations (Todt et al., 2005; Eppsteiner and Smith, 2011). Results of the present study suggest this relative *connexin* insensitivity in vestibular organs compared to the cochlea could be due to differences in the role of intercellular calcium signaling during development.

Intracellular calcium transients occurred in different SCs at sequential times, however these cells were rarely adjacent to each other (Figure 3 and Supplement Videos). If temporal correlations between transients occurring in SCs were causally related it would require signaling between distant SCs. One hypothesis is that utricular SC calcium transients could be modulated by efferent neural activity acting primarily on HCs and afferent terminals (Holt et al., 2015, 2017) and subsequently signaling SCs. Synaptic inputs from efferent neurons are active in the cochlea during this developmental period and are likely critical for maturation of synaptic function, tonotopy and hearing (Katz et al., 2004; Johnson et al., 2013). Extension of this concept to the utricle is supported by the present data showing that exogenous application of the efferent transmitter ACh synchronized SC calcium transients beginning in aged P3-P5 mice (Figure 5). The dendritic fields of vestibular efferent neurons in the utricle are morphologically complex, making synaptic contacts with HCs and afferent terminals across broad regions. Hence, activation of a single efferent neuron could potentially synchronize SC activity and HCs across diverse spatial locations in the epithelium. This mechanism hypothetically has potential to close the loop between afferent inputs to the CNS and efferent feedback to the sensory organ—closed loop feedback that might be a general developmental principle organizing the auditory and vestibular systems from the sensory organs to the CNS and back again.

Mice aged P8 and older (Figure 7D) continued to show “spontaneous” ΔF/F [Ca^2+^]_i_ transients in SCs. Spontaneous [Ca^2+^]_i_ activity suggests SCs in adult utricles exhibit whole cell transients as part of their mature physiological function, perhaps serving roles similar to astrocytes in the CNS (Dani et al., 1992; Agulhon et al., 2008; Bazargani and Attwell, 2016; Shigetomi et al., 2016). Adult vestibular organs express both nicotinic nAChRs and muscarinic mAChRs that together underlie both inhibitory and excitatory responses to activation of the efferent vestibular nucleus or to the application of ACh (Guth et al., 1994, 1998; Elgoyhen et al., 2001; Holt et al., 2003, 2017; Boyle et al., 2009; Goldberg, 2012; Lee et al., 2017; Parks et al., 2017). nAChRs are calcium permeable and their activation leads to short local calcium increases, while mAChRs are metabotropic and can evoke long lasting responses. The G5 indicator used in the present study is has kinetics likely unable to track localized [Ca^2+^]_i_ events associated with nAChR activation, but is well suited to examine long lasting G protein coupled [Ca^2+^]_i_ transients associated with mAChR activation. Some of the most striking [Ca^2+^]_i_ transients observed in the present report were ACh evoked, with increases in [Ca^2+^]_i_ largely blocked by 50μM 2-APB and/or ATR (Figures 6, 7; Supplement Video 7). ACh sensitive cells in mature utricles contacted type I hair cells and had morphologies consistent with calyx afferent endings. The long-lasting ACh-evoked [Ca^2+^]_i_ transients in mature utricles in the present report had durations similar to mAChR dependent electrophysiological responses reported previously in calyx bearing afferents (Holt et al., 2017), suggesting a potential role for CICR in modulating KCNQ channels during activation of the efferent system in calyces.

The fact that the Gad2-IRES-Cre used in the present study drove expression preferentially in a distinct subset of type I hair cells could have important implications, but was not addressed in the present study. Although there is evidence of glutamate decarboxylase isoform GAD67 expression in vestibular SCs (Tavazzani et al., 2014), the pattern of tdT-G5 expression observed in the present study suggests embryonic transcriptional regulation of Gad2 in a subset of type I hair cells. Previous studies indicate that GAD65 has multiple transcription start sites (Skak and Michelsen, 1999), and that GABA might act as a morphogen during development (Owens and Kriegstein, 2002). It is therefore difficult to draw direct conclusions of the Gad2 related transcriptome from the complex expression pattern in the present transgenic mouse. Nevertheless, the pattern observed at birth and into adulthood suggests the role of Gad2 in extrastriolar type I HCs likely differs significantly from type II HCs and from striolar type I HCs. While the primary vesicular transmitter in HCs is glutamate, the expression pattern provides further evidence that the GAD65-dependent molecular machinery responsible for synthesizing GABA is present (Usami et al., 2001; Moser et al., 2006; Meza, 2008). Furthermore, there is evidence that GABA may be utilized as a secondary intercellular transmitter in a subset of vestibular HCs in toadfish (Holstein et al., 2004), but evidence of similar mechanisms have not been reported in mammals to date. Although present results are suggestive, examination of the role of GAD65 in vestibular organs remains an important subject for future research. Irrespective of the involvement of Gad2, using its transcriptional machinery to drive expression of the genetically encoded calcium indicator G5 enabled examination of [Ca^2+^]_i_ transients in early postnatal developmental stages in the present study.

The [Ca^2+^]_i_ transients in the present report are limited by the speed and dynamic range of the G5 transgenic reporter. The dissociation constant, *K*_*d*_, of G5 is approximately 460 nM, similar to Fluo-4 (350 nM) and useful for [Ca^2+^]_i_ imaging in SCs, HCs, and neurons but not always ideal (Oliver et al., 2003; Akerboom et al., 2012; Spinelli and Gillespie, 2012). Cell dependent differences in resting [Ca^2+^]_i_ influence optimum *K*_*d*_, and differences in level of expression contribute to gain, both influencing the quantitative relationship between ΔF/F_0_ and changes in [Ca^2+^]_i_. Because of this, G5 ΔF/F_0_ in the present study reports the spatial extent and kinetics of [Ca^2+^]_i_ modulation without addressing quantitative magnitude. The sampling rate used in experiments described here was 100 ms per frame, nearly equivalent to the rise time and faster than the decay time of the G5 indicator (Akerboom et al., 2012; Chen et al., 2013). Although present results demonstrate postnatal maturation of spontaneous calcium transients and development of ACh sensitivity, expanding these studies with additional techniques *in vivo* will be important to fully elucidate [Ca^2+^]_i_ activity during development.

It's important to emphasize that spontaneous activity comprised of coordinated [Ca^2+^]_i_ transients is essential to the developing vestibular nervous system, shown previously to extend through the CNS as well as in the developing retina and cochlea (Wong et al., 1995; O'Donovan, 1999; Brandt et al., 2003; Tritsch et al., 2007; Ackman et al., 2012). Data in the present report demonstrate spontaneous activity is present in mouse utricular sensory HCs and that these cells are likely undergoing differentiation at birth, before the onset of mature vestibular function. Spontaneous activity in HCs subsides by P2, while spontaneous activity in SCs develops during the first two weeks and is maintained into old age. Present results further demonstrate the development of long-lasting, ATR sensitive, ACh-evoked transients during this same time period putatively associated with maturation of mAChR-dependent vestibular action on calyx-bearing afferent responses.

## Ethics Statement

This study was carried out in accordance with the recommendations of the University of Utah Institutional Animal Care and Use Committee (IACUC). The protocol was approved by the University of Utah IACUC.

## Author Contributions

HH and RR designed experiments. HH, LP, and RR performed experiments. MF designed and built components of the swept field confocal microscope. HH and RR analyzed and processed the data. HH and RR wrote the manuscript. LP, MF, HH, and RR proofread and edited the manuscript.

### Conflict of Interest Statement

The authors declare that the research was conducted in the absence of any commercial or financial relationships that could be construed as a potential conflict of interest.

## Supplementary Material

Supplement Video 1 Supplement to Figure 2 in the parent paper. Time sequence of images showing spontaneous [Ca^2+^]_i_ transients in extrastriolar type I utricular hair cells (HCs) at P1. Resting G5 fluorescence (blue) is merged with G5 ΔF/F fluorescence modulation (green) reporting changes in [Ca^2+^]_I_ (green).

Supplement Video 2 Supplement to Figure 3 in the parent paper. Time sequence of images showing spontaneous [Ca^2+^]_i_ transients in utricular supporting cells (SCs) at P3. Resting G5 fluorescence (blue) is merged with G5 ΔF/F fluorescence modulation (green) reporting changes in [Ca^2+^]_i_ (green). Image plane cut through the epithelium at a shallow angle to image deep in the tissue through the based of supporting cells (upper left) and near the apical surface (lower right). Note high levels of resting G5 fluorescence in type I HCs and large numbers of spontaneous transients in SCs.

Supplement Video 3 Supplement to Figure 3 in the parent paper. Same format as Supplement Video 2, but from a different animal.

Supplement Video 4 Supplement to Figure 3 in the parent paper. Same format as Supplement Video 2 in alternative tissue with ΔF/F in multiple cell types and regions.

Supplement Video 5 Supplement to Figure 6 in the parent paper. Time sequence of images showing spontaneous [Ca^2+^]_i_ transients in utricular SCs at P8. G5 ΔF/F fluorescence modulation reporting changes in [Ca^2+^]_i_ (green) and resting G5 fluorescence (blue) are merged.

Supplement Video 6 Supplement to Figure 6 in the parent paper. Time sequence of images showing 100 μM ACh evoked [Ca^2+^]_i_ transients in utricular HCs, calyces (C), and unidentified small domains (R) at P8. G5 ΔF/F fluorescence modulation reporting changes in [Ca^2+^]_I_ (green) and resting G5 fluorescence (blue) are merged. Image blurring following ACh application is an artifact caused by motion as the ACh solution washed over the epithelium.

Supplement Video 7 Supplement to Figure 7 in the parent paper. Time sequence of images showing spontaneous and 100 μM ACh evoked [Ca^2+^]_i_ transients at P533. G5 ΔF/F fluorescence modulation reporting changes in [Ca^2+^]_i_ (green) and resting G5 fluorescence (blue) are merged.
